# Evolving Belief, Evolving Minds: Evolutionary Insights Into the Development and Functioning of Human Society

**DOI:** 10.3389/fnbeh.2022.928297

**Published:** 2022-06-22

**Authors:** Agustín Fuentes

**Affiliations:** Department of Anthropology, Princeton University, Princeton, NJ, United States

**Keywords:** evolution, neurobiology, human mind, genus *Homo*, belief

Belief involves the ability to think beyond what is here and now and develop mental representations in order to see and feel and know something not immediately present to the senses, and invest in that something so that it becomes one's reality (Fuentes, 2019). Belief involves mental activity constituted by neural circuits in the brain (Boyer, 2003), but it is more than that. Belief involves the human ability to draw on cognitive and social resources, histories and experiences, and combine them with imagination to produce neurobiological, physiological, mental and social experience. Belief is a capacity, which may include manifestations of a mental state or attitude involving the appraisal of a proposition, but is not simply this particularly cognitively complex human ability of perceptive and affective information processing (e.g. Seitz and Angel, 2020). Nor is belief solely a property arising from the human capacity for extensive shared agency and shared intentionality (e.g. Tomasello, 2019), although both of those processes form aspects of the human capacity for belief. The capacity for belief enables the human to commit wholly and fully to an idea, a sensation, a concept such that it structures perceptual and experiential processes. Beliefs and belief systems permeate contemporary human neurobiologies, bodies, and ecologies, acting as dynamic agents in evolutionary processes and playing core roles in structuring human societies and the human mind (Stotz, 2010; Downey and Lende, 2012; Han, 2017; Fuentes, 2019; Seitz and Angel, 2020).

Belief is not an ‘emergent property’, something ephemeral floating above the material reality of being human. It is a central component of the human experience. The ability to believe is part of the human system similar to the way that fingers are part of our arms and hands. Fingers are core aspects of human anatomy, modified over evolutionary time dramatically expanding our options for interacting with the world and each other. In humans, mammalian and then primate limbs were shaped and altered over evolutionary time so that their ends contain structures (prehensile digits and hands with precision grips) expanding the capacities for engagement with, and manipulation of, the world. The capacity for belief is similar: it expands human cognitive, sensory and perceptual dynamics and is critical in the human ability to engage with and shape the world.

In an evolutionary context, beliefs provide for both novel alterations and continued coherence in the human niche. In this brief essay I outline key elements in human evolutionary history that facilitated the emergence of the capacity for belief and suggest that beliefs act as core niche constructive processes in the development of the human mind.

## Evolutionary Context and History

**Figure 1:**
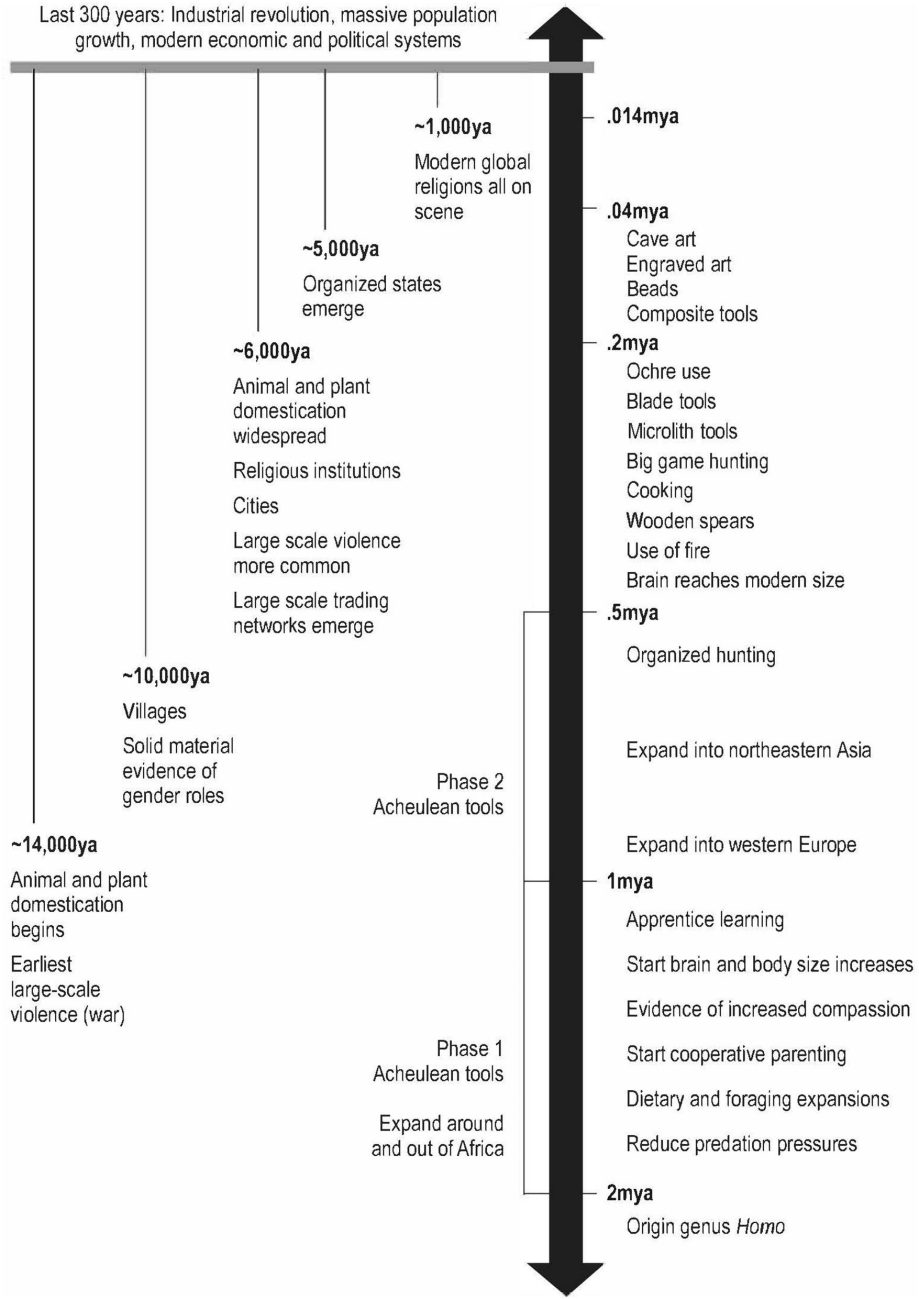
A summary overview of the many key capacities, events and processes in the human niche dated to their first appearances in the fossil and archeological record. Reproduced from Fuentes, 2018, 2019, with permission from University of Chicago Press and Yale University Press.

A niche is the structural, temporal, and social dynamic in which a species exists. The niche involves the interfaces between individuals and space, structure, climate, nutrients, and other physical and social factors as a dynamic set of interacting processes (Wake et al., 2009). Over the last two million years members of the genus *Homo* (humans) underwent significant changes via the emergence of a distinctively human niche. Relative to other hominins, *Homo* underwent specific morphological changes alongside significant behavioral, ecological and cognitive shifts as they forged and were shaped by this human niche (Fuentes, 2015; Marks, 2015; Antón and Kuzawa, 2017; Kissel and Fuentes, 2021). During this time core human patterns emerged including: hyper-cooperation and complex collaboration in social interactions and material technologies; substantially extended childhood development and complex caretaking behavior; intricate and diverse foraging and hunting patterns involving complex technologies, behavior and communication; novel and dynamic material and symbolic cultures eventually resulting in complex cognitive and material meaning-making processes; emergence of exchange networks and increasingly dynamic intergroup relations; and increasingly complex communication and information sharing, eventually resulting in language (Foley, 2016; Fuentes, 2017, 2018; Galway-Witham et al., 2019) (Figure 1).

Across the last million years there were many morphologically and behaviorally diverse populations of the genus *Homo* occupying and shaping the human niche, initially across Africa and Eurasia and eventually into Australasia, the Americas and multiple islands across the planet. The taxonomic distinctions between these populations are far from clear. Some argue for multiple species and others for many subspecies, with others suggesting that it is not currently possible to determine the correct number and types of taxa within the genus *Homo* (Schwartz and Tattersall, 2015; Wood and Boyle, 2016). Given the morphological and ecological diversity, and the multiple tool technologies and lifeways evident across this period it is clear that there were many successful ways to navigate the human niche and that they all were intricately connected to, and stemming from, an evolving cognitive capacity setting the stage for the contemporary human mind. Contemporary *Homo sapiens* are inheritors of a diversity of biological and cultural histories facilitated by the dynamics of the human niche (Kissel and Fuentes, 2021).

Current integrative approaches to human evolution emphasize mutual mutability between agents, bodies, collective action, social perceptions, and the roles of experiences, cultures and institutions in structuring human behavior (Fuentes, 2009, 2017; Marks, 2012; Fry, 2013; Kim and Kissel, 2018; Seitz et al., 2018; Sykes, 2020; Kissel and Fuentes, 2021; e.g. DeSilva, 2021). Such complex and multifarious dynamics model interfaces of ecological, behavioral, cultural and cognitive processes as core in the human niche enabling conjectures about the processes at play in the emergence of distinctively human cognition and thought, a “human mind.”

In previous work (Fuentes, 2015, 2016, 2017) I've argued for envisioning the human niche as encompassing individual bodies and their evolutionary histories and the patterns and dynamics of interactions within social groups, interactions among/between social groups, and at the community/population levels all within an interactive dynamic with local ecologies (see also Whiten and Erdal, 2012; Foley, 2016). In such a model, evolutionary processes exert pressures at various nodes in the system and responses to those pressures emerge at individual, group, and community levels. The human niche is a dynamic produced by proactive and reactive responses to social and ecological pressures and contexts at various levels creating local and regional ecologies of interactive material, social, cognitive, and historical aspects that flow from one generation to the next; it creates a shared ecology across time and space, the cultural context in which humans evolve (Henrich, 2016; Fuentes, 2017; Laland, 2017; Boyd, 2018). In the development of that human niche the capacity for belief emerged as a significant component creating a dynamic suite of affordances and constraints on human lives facilitated through human cognition, perception, and thought. The evolution of the human niche then, included the emergence of a shared imagination and a suite of distinctive socio-cognitive processes (Whiten and Erdal, 2012; Tomasello, 2014; Fuentes, 2017; Laland, 2017) and a ubiquitous semiotic ecosystem (Deacon, 2016) as central to the context in which humans evolve.

## Meaning Making, Culture, and Contemporary Human Cognition

The environment humans make for themselves is created through their symbol using ability, their capacity for abstraction. The symbols, the ideas, are created in the mind... but the human animal learns not only to create them, but to project them onto the external world, and there transform them into reality. –Montagu (1965), The Human Revolution [1965:2–3]

The patterns and processes of contemporary human cognition and culture, the human perceptual landscape and core facets of human minds, emerged alongside the processes of toolmaking, foraging, caretaking, the control of fire, the creation of symbolic materials, and the ecological expansion of humans across the planet. This ongoing dynamic, the feedback between neural and behavioral plasticity, laid the neurobiological, social, and ecological foundations in human populations for a particularly complex cognition, and for belief (Deacon, 1997; Fuentes, 2019; Tomasello, 2019; Corbey, 2020). The ratcheting up of social and ecological complexity, combined with increased interactions among populations of the genus *Homo*, particularly over the last 200,000 to 500,000 years, created opportunities for the connections and exchanges between groups and populations that enabled shared beliefs, and eventually belief systems, to emerge (Galway-Witham et al., 2019; Kissel and Fuentes, 2021). The last few hundred thousand years offer material evidence for an increase in, and eventual ubiquity of, meaning-making, art and symbol in human populations (Malafouris, 2013; Deacon, 2016; Roberts, 2016; Fuentes, 2017; Sykes, 2020). Across this process humans developed a capacity for imagination and conceptual innovation. These cognitive processes entailed the emergence of two significant patterns. First, the imagining of novel items and/or representations and either making them or altering other things to become them. Such a capacity appears in a limited form in other animals but becomes permanently and ubiquitously part of the human niche by the middle to late Pleistocene. Second and drawing on the first, over the last few hundred thousand years of our history, as part of our intensive communicative and semiotic capacities, humans began creating explanations of widely observable phenomena such as death, the behavior of other animals, weather, or the sun and moon. They did not, for example, simply connect clouds, thunder, rain, and floods, they also developed explanations for why these things happen (Deacon, 1997, 2016; Tomasello, 2014; Henrich, 2016; Fuentes, 2017, 2018, 2019; Kissel and Fuentes, 2017). This capacity is what Bloch (2008) refers to in arguing that over evolutionary time humans went from socially complex transactional beings (like most social mammals and other primates) to groups of organisms who exist simultaneously in both transactional and transcendent realities, and who use imagination and belief to reshape themselves and the world around them (Fuentes, 2019).

## Human Culture/Human Mind

While many organisms have cultures (Whiten, 2021), human culture is demonstrably distinctive. Human culture affects the way that humans do almost everything: fighting, eating, reproducing, innovating, interacting, cooperating, perceiving, making and using technology, expressing ourselves, experiencing emotions, and a host of other cognitive and behavioral processes and events. Culture makes human reason, human being, possible; it forms the central facet of the human niche (Tomasello, 2014; Laland, 2017). Yet individual cultures constrain as much as they enable. Cultures shape social processes and outcomes as well as individuals' development. Cultural contexts, the “webs of significance” that are symbolic meaning, are both materially and perceptually real for the people within them and thus structurally relevant to, and affected by, evolutionary processes and societal processes. When something happens – an action, observation, or experience – our cultural context helps give it meaning, and our participation in that culture enables us to interact with that meaning, making the engagement dynamic and malleable. So, if culture has meaning, then the symbols, ideals, and traditions human participate in come ready-made with relevance and connection to our personal schemata; they make sense to us and shape how we interact with the world. When culture becomes a species' capacity and necessity, as it is for *Homo sapiens*, understanding the mechanisms by which cultural processes evolved, how they function and how such function impacts members and populations of that species itself is of primary interest in any evolutionary narrative of the mind.

For example, a stone tool is not relevant to human evolution simply as the combination of a person altering and using a shaped stone, but rather requires the fact that a person has a set of beliefs, or concepts, of a tool to begin with. The stone object is given shape but also a functional capacity in affecting the world by being transformed from stone to tool, not just through mechanical modification, but also by an understanding about “tool” as a concept. Such assemblages of practical and conceptual processes are a cognitive outcome of evolved capacities in the human niche. A human with the tool concept, and beliefs about the tools themselves, is not constrained by existing tools or materials when novel challenges arise. Rather they can try to innovate and find and modify a stone, or other material, into a novel or altered tool for the job. Likewise, beliefs can shape how social interactions and behavior impact bodies. The contemporary belief of an infant as a fragile (or not) body affects adult handling of infants in ways that influence the maturation processes in a child's motor system, leading to differences in the attainment of landmark events in motor development by working through parental behavior on developmental pathways (Hopkins and Westra, 1990). On a broader populational scale, a shared cultural belief in monotheism can affect social organization and has significant impacts on human reproduction, phenotype, or functioning. It can be linked, for example, to entrenched social inequality such that it makes hierarchy and differential resource distribution more likely to occur, and it increases the likelihood of large state formation or endurance (see Norenzayan, 2013; Henrich, 2020).

Cultural beliefs are important because they fundamentally and reliably change humans' relationships to our environments, the resources at our disposal (e.g., tools, senses, communication), and the conditions of our maturation (the developmental niche), which can have both intra- and intergenerational impact (Seitz et al., 2018; Fuentes, 2019). They are a fundamental part of the niche into which humans born and through which they will interact with the world and other people. Rather than rehashing either side of well-worn debates about the relative importance or contribution of biological and cultural processes, it is evident that the human experience is composed of interacting, co-determining elements of both. And that this process evolved as a central component of the human niche. Human neuroanatomy makes experience material—neural systems adapt through long-term refinement and remodeling, which leads to learning, memory, maturation, which structure perception and affect the creation of beliefs. Through systematic change in the nervous system, and immersion in cultural contexts, humans learn to orchestrate themselves. Cultural concepts and meanings become anatomy (Downey and Lende, 2012). Beliefs infuse human minds, bodies, and ecologies, creating dynamic perceptual and interpretative assemblages that can act either as robust ‘enculturalizing’ forces in human social systems/socioecologies (our cultures) or disrupt them, facilitating new and/or modified dynamics in perceptual and cultural processes. Therefore belief, and its related cognitive processes and their evolutionary history, matters in assessing human behavior and experience; belief shapes the human mind, past, present, and future.

## Author Contributions

AF conceived, wrote, and revised all aspects of this article.

## Conflict of Interest

The author declares that the research was conducted in the absence of any commercial or financial relationships that could be construed as a potential conflict of interest.

## Publisher's Note

All claims expressed in this article are solely those of the authors and do not necessarily represent those of their affiliated organizations, or those of the publisher, the editors and the reviewers. Any product that may be evaluated in this article, or claim that may be made by its manufacturer, is not guaranteed or endorsed by the publisher.
